# Targeting Autophagy for Acetaminophen-Induced Liver Injury: An Update

**DOI:** 10.3390/livers4030027

**Published:** 2024-08-14

**Authors:** Kaitlyn Hinz, Mengwei Niu, Hong-Min Ni, Wen-Xing Ding

**Affiliations:** 1 Department of Pharmacology, Toxicology and Therapeutics, University of Kansas Medical Center, Kansas City, KS 66160, USA; 2 Department of Internal Medicine, University of Kansas Medical Center, Kansas City, KS 66160, USA

**Keywords:** mitophagy, NRF2, p62/SQSTM1, TFEB

## Abstract

Acetaminophen (APAP) overdose can induce hepatocyte necrosis and acute liver failure in experimental rodents and humans. APAP is mainly metabolized via hepatic cytochrome P450 enzymes to generate the highly reactive metabolite *N*-acetyl-*p*-benzoquinone imine (NAPQI), which forms acetaminophen protein adducts (APAP-adducts) and damages mitochondria, triggering necrosis. APAP-adducts and damaged mitochondria can be selectively removed by autophagy. Increasing evidence implies that the activation of autophagy may be beneficial for APAP-induced liver injury (AILI). In this minireview, we briefly summarize recent progress on autophagy, in particular, the pharmacological targeting of SQSTM1/p62 and TFEB in AILI.

## 1. Introduction

Acetaminophen (APAP) is a widely used analgesic and antipyretic drug in the United States [1–3]. However, APAP overdose can lead to acute liver failure, which is responsible for nearly half of drug-induced liver injury cases and is a leading cause of liver failure in Western countries [4–7]. Currently, the most commonly used treatment for acetaminophen-induced liver injury (AILI) is N-acetylcysteine (NAC), which restores glutathione (GSH) levels in the liver. However, the time window for treatment with NAC is limited. Therefore, there is an urgent need to develop novel treatments for AILI [8].

Several distinctive phases of liver pathogenesis in AILI have been documented, including the early metabolic phase, injury phase, and liver repair/recovery phase [9]. In the early metabolic phase, APAP is mainly metabolized by conjugation with glucoronidate and sulfate in the liver via phase II enzymes, and only 5–9% of APAP is metabolized via cytochrome P450 enzymes, primarily, cytochrome P450 2E1 (CYP2E1) and CYP1A2, to form a highly reactive metabolite, *N*-acetyl-*p*-benzoquinone imine (NAPQI), which is detoxified by hepatic GSH. However, excessive NAPQI can covalently bind to intracellular proteins to form cytosolic and mitochondrial APAP protein adducts (APAP-adducts) leading to mitochondrial damage and increased oxidative stress, which are followed by the activation of c-Jun N-terminal kinase (JNK) and release of mitochondrial endonuclease G and an apoptosis-inducing factor to trigger DNA fragmentation and hepatocyte necrosis (necrotic injury phase) [10–14]. After the initial phase of necrotic injury, the liver undergoes several adaptive responses in the repair/recovery phase. These responses include the innate immune response and blood coagulation events, which can either worsen or alleviate AILI during the recovery phase [15–17]. Macrophages from the bloodstream can help resolve the injury by eliminating dead cells and inducing the death of neutrophils [18]. Recent evidence shows that during the late recovery phase of AILI, increased hepatic platelet aggregation can inhibit liver regeneration. This happens because of the increased secretion of the platelet adhesive protein, Von Willebrand factor (VWF), in the liver during AILI [19].

Autophagy, a lysosomal degradation pathway, removes APAP-adducts and damaged mitochondria as another line of adaptive protective response against AILI [20–23]. Autophagy can selectively remove protein aggregates and damaged/excess organelles, and is mediated by a group of proteins called autophagy receptor proteins, including SQSTM1/p62 (hereafter referred to as p62), optineurin, NDP52, NIX, BNIP3, FUNDC1, and prohibitin 2 [24–27]. As lysosomes sit at the end for the execution of autophagic degradation via lysosomal enzymes, the number and function of lysosomes are critical for completing the autophagy process. Increased lysosomal biogenesis is necessary to meet the need for the fusion with autophagosomes and subsequent autophagic degradation. The transcription regulation of lysosome biogenesis genes is mediated by transcription factor EB (TFEB), a basic helix–loop–helix leucine zipper transcription factor belonging to the coordinated lysosomal expression and regulation (CLEAR) gene network [28]. Notably, TFEB also regulates mitochondrial biogenesis by directly regulating the expression of PGC-1α, a key transcription coactivator in mitochondrial biogenesis [29,30]. The purpose of this review is to briefly summarize recent progress on manipulating p62 and TFEB in AILI, and hopefully stimulate more future studies to identify novel p62 and TFEB agonists to treat AILI.

## 2. Targeting p62 for AILI

p62 is a multidomain scaffold protein that plays a crucial role in various cellular processes, including signal transduction pathways for cell survival, cell death, and antioxidant stress responses [31–34].

At its N-terminus, the PB1 (Phox/Bemp1) domain of p62 is essential for its localization to the autophagosome formation site [35]. The PB1 domain mediates p62 self-oligomerization, and it also interacts with other PB1-containing proteins, such as atypical protein kinase Cζ (PKCζ) and neighbor of BRCA1 gene 1 (NBR1) [36,37]. Mitogen-activated protein kinase kinase kinase 3 (MEKK3) also contains a PB1 domain, which forms a heterodimer with the PB1 domain of p62 and binds to TRAF6, a lysine 63 (K63) E3 ligase, to trigger nuclear factor-κB (NF-κB) activation [38]. The PB1 domain is followed by a ZZ-type zinc finger (Znf) domain, which is required for efficient starvation-induced autophagy in mouse embryonic fibroblasts (MEF) [39], and it also binds to receptor interacting protein (RIP) to regulate NF-κB activation [40].

The N-end rule pathway is a proteolytic system in which single N-terminal amino acids serve as determinants of degrons called N-degrons [41]. This pathway helps to break down and dispose of certain proteins that are misfolded or otherwise damaged. N-degrons can be created by cutting the protein at the end and modifying the remaining N-terminal residues through reactions such as deamidation, oxidation, and arginylation. The main degron is Nt-Arg (N-terminal Arginine), which is created by adding L-Arg to Nt-Asp or Nt-Glu using Arginyl-tRNA-protein transferase 1. Proteins with Nt-Arg are recognized and bound by N-recognins, which contain a UBR box, and are then broken down into shorter peptides by the ubiquitin proteasome system (UPS). Recent evidence suggests that the ZZ domain of p62 is a structural and functional counterpart of the UBR box in N-recognins in the UPS-linked N-end rule pathway for autophagic degradation of Nt-arginylated substrates, including protein aggregates, and promotes mitophagy and ER-phagy [42–44].

Next to the ZZ domain is the TRAF binding (TB) domain, which also activates NF-κB via interacting with ubiquitin E3 ligase TRAF6 [33,45]. Additionally, p62 interacts with RAPTOR via the region between the ZZ and TB domains to activate the mechanistic targets of rapamycin complex 1 (mTORC1) [46].

p62 directly binds to LC3 through the LC3-interacting region (LIR), and thus acts as an autophagy receptor protein for selective autophagy [47,48]. Followed by the LIR domain is a Kelch-like ECH-associated protein 1 (KEAP1)-interacting region (KIR) that binds to KEAP1 and drives KEAP1 degradation by selective autophagy, resulting in nuclear factor erythroid 2-related factor 2 (*nfe2l2* or NRF2) activation via the noncanonical KEAP1-NRF2 pathway [49–52]. p62 and KEAP1 positive aggregates have been observed in autophagy-deficient mouse livers, causing the persistent activation of NRF2 in the liver [53–55]. Finally, at the C-terminus of p62, the ubiquitin-associated (UBA) domain binds to ubiquitin-labeled proteins or damaged organelles and leads them into the autophagosome for degradation [33,56].

Recent studies demonstrate that autophagy, a lysosomal degradation pathway, protects against AILI by promoting the removal of APAP-adducts, damaged mitochondria, and stressed ER [20–23,44,57]. Several pieces of evidence support that autophagy may selectively remove APAP-adducts involving p62. First, the levels of hepatic APAP-adducts reach higher levels at the metabolic and injury phases but decline at the late recovery phase, suggesting the activation of an APAP-adduct removal mechanism at the late recovery phase. Interestingly, hepatic levels of p62 also increased after APAP treatment for 24 h, which is inversely correlated with the decreased hepatic levels of APAP-adducts [58]. Second, APAP-adducts colocalize with GFP-LC3 positive autophagosomes, and this colocalization is enhanced in the presence of a lysosomal inhibitor which raises lysosomal pH and blocks degradation [23]. Third, isolated autophagosomes and lysosomes from APAP-treated mouse livers contain APAP-adducts [23]. Fourth, APAP-AD are ubiquitin-positive and colocalized with a lysosomal marker LAMP1. Fifth, the levels of APAP-adducts increase in APAP-treated primary hepatocytes with p62 knockdown via an adenovirus shRNA treated with APAP, and in p62 whole body knockout (KO) mice at 24 h after APAP treatment [23,58]. Sixth, the pharmacological inhibition or activation of autophagy increases or decreases hepatic levels of APAP-adducts, respectively [20,23]. While the above evidence supports the role of p62 in the selective removal of APAP-adducts by autophagy, it remains unclear how p62 would be recruited to APAP-adducts. The UBA domain of p62 is likely critical as it can mediate its binding with ubiquitin-positive APAP-adducts. Indeed, in NAPQI-treated Hep3B cells, mutant p62 with UBA deletion failed to be recruited to the mitochondria, although its role in the removal of APAP-adducts was not determined [44]. Future studies to investigate the role of UBA-deleted p62 mutants in the removal of APAP-adducts are needed to test this hypothesis. Another important unanswered question is how APAP-adducts become ubiquitin-positive. Whether this would involve a specific E3 ligase remains to be determined.

As discussed earlier, the p62 ZZ domain interacts with Nt-Arg residues. p62 can serve as the N-recognin to facilitate p62 in complexes with cargoes, promoting selective autophagic cargo degradation [41–43]. Based on this principle, 3D structured modeling of p62 and screening a library of 540,000 compounds have led to identifying small molecules of p62 agonists that enhance its selective autophagic degradation [59,60].

Among these p62 agonists, YTK-2205 has been shown to protect against AILI in mice by promoting hepatic mitophagy, ER-phagy, and autophagy of ubiquitin protein aggregates without affecting the NRF2 pathway. While the YTK-2205 treatment offered marked protection against AILI based on the decreased serum alanine aminotransferase and hepatic necrotic areas in mice, the protection was lost when YTK-2205 was given 1.5 and 3.5 h post-APAP injection [44]. Therefore, the use of YTK-2205 for the clinical treatment of AILI patients will be limited. However, a combined treatment of YTK-2205 with NAC may offer better beneficial effects than NAC or YTK-2205 administration alone, which should be tested in animal models in the future. While YTK-2205 promotes the recruitment of p62 to ubiquitinated mitochondria and ER, it remains unclear how increased ubiquitinated mitochondria and ER would occur after APAP treatment [44]. We previously showed that APAP treatment increased mitochondrial PARKIN translocation and subsequently increased levels of mitochondrial ubiquitin and p62, and that both PARKIN and PINK1 are necessary for mitophagy in AILI [21,22]. Small molecule activators of PINK1 or PARKIN are currently under preclinical development [61]. It would be very interesting to test the beneficial therapeutic effects of the combination of PINK1 and PARKIN activators with YTK-2205 in AILI in the future.

In addition to its role in selective autophagy, p62 may also protect against AILI via its noncanonical NRF2 activation. As discussed earlier, p62 directly interacts with KEAP1 through KIR to activate NRF2 in a nontraditional way [50,52,62]. NRF2 activation then leads to the expression of genes that synthesize GSH and antioxidants as well as detoxifying enzymes, glutamate cysteine ligase (GCL), and NADPH quinone dehydrogenase 1 (NQO1), which protect against AILI [63,64]. In liver-specific Atg5 KO mice, the accumulation of p62 in the liver leads to persistent NRF2 activation, resulting in increased levels of NQO1 and better recovery of GSH, which in turn attenuates AILI [54].

In addition to its role in protecting against AILI, p62 also regulates the activation of other proteins, such as NF-κB and mTORC1, which may be involved in liver regeneration after an overdose of APAP, particularly, in the late recovery phase of AILI [33,37,65]. Indeed, APAP treatment activates mTORC1 at 24 h, which is associated with increased hepatic p62 and cell proliferation in mouse livers. In contrast, p62 KO mice showed decreased mTORC1 activation and cell proliferation with aggravated liver injury at 24 h after APAP treatment. While it appears that p62 is protective at the injury phase of AILI, p62 may halt the recovery phase of AILI as p62 KO mice recovered better than the wild-type mice at 48 h.

As mentioned earlier, increased hepatic VWF and platelet accumulation impair liver regeneration [19]. The p62 KO mice demonstrated a decrease in hepatic VWF and platelet aggregation, which are associated with increased cell proliferation and improved liver injury after 48 h of APAP treatment [58]. While it remains unclear how p62 would affect VWF and platelet recruitment to the liver, these observations clearly support a dual role of p62 in AILI in which p62 inhibits the injury phase of AILI by increasing the selective removal of APAP-adducts and damaged mitochondria through autophagy but impairs the recovery phase of AILI, likely by enhancing hepatic blood coagulation.

**Figure 1.**
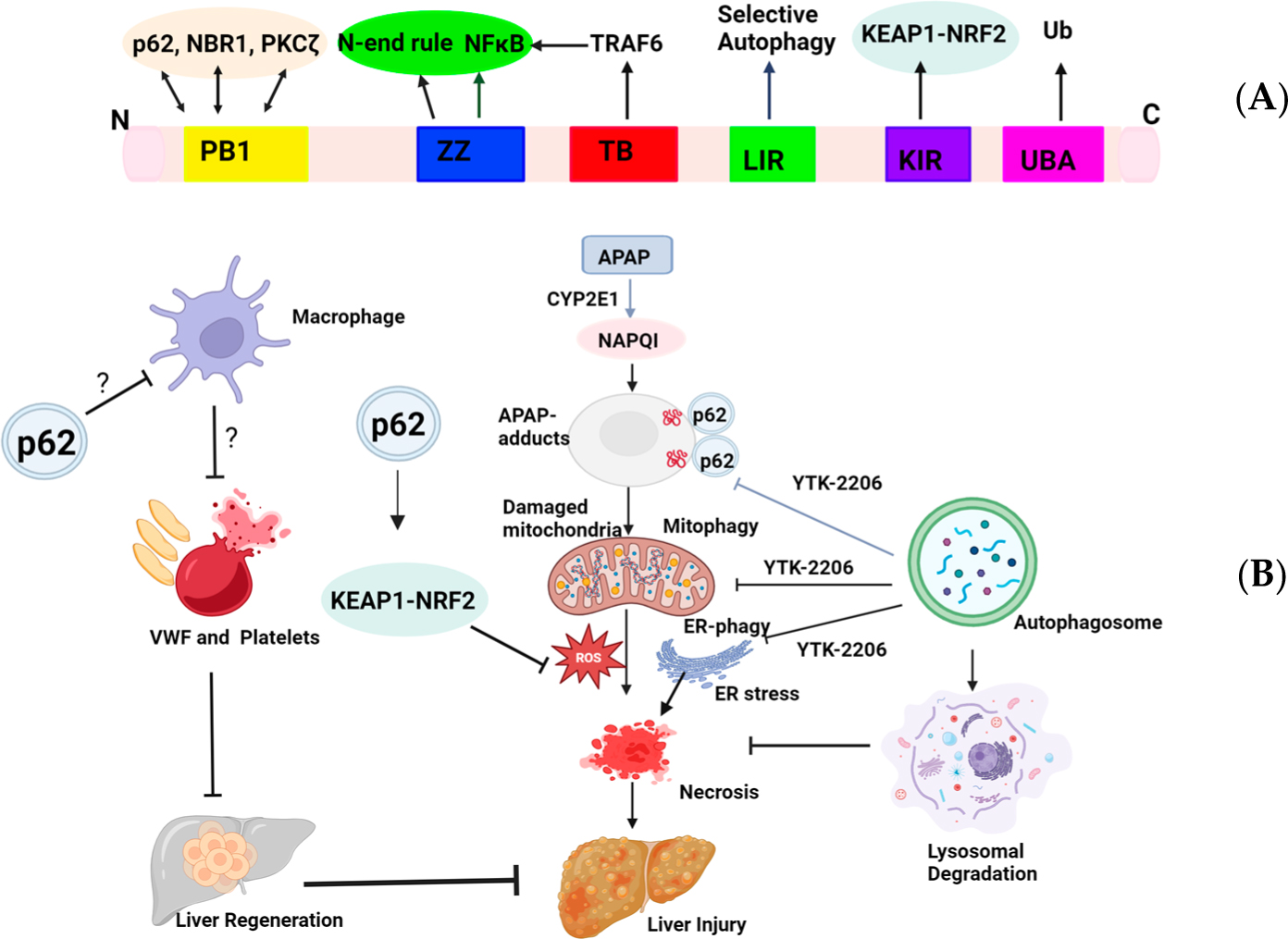
A proposed model for targeting p62 in acetaminophen-induced liver injury (AILI). (**A**) Schematic domain structure of sequestosome 1 (SQSTM1/p62). The Phox and Bem1 (PB1) domain sits near the N-terminus of p62 and interacts with PB1-containing proteins, including p62 itself, neighbor of *BRCA1* gene 1 (NBR1), and atypical protein kinase Cζ (PKCζ), to form homooligomers or hetero-oligomers. The ZZ-type zinc finger (ZZ) mediates NF-κB activation and N-end rule degradation. The tumor necrosis factor receptor-associated factor 6 (TRAF6)-binding domain (TB) interacts with TRAF6 for NF-κB activation. The LC3-interacting region (LIR) domain interacts with the microtubule light chain 3 (LC3) protein to trigger selective autophagy. The Kelch-like ECH-associated protein 1 (KEAP1)-interacting region (KIR) binds to KEAP1 for the noncanonical-nuclear factor erythroid 2-related factor 2 (NRF2) activation. The ubiquitin (UB)-associated (UBA) domain sits close to the C-terminus that binds to ubiquitinated proteins. (**B**) A proposed model for p62 in selective autophagy in AILI. APAP metabolism via cytochrome P450 2E1 (CYP2E1) generates NAPQI that forms APAP-adducts that are ubiquitinated by unknown mechanisms. APAP also induces mitochondria ubiquitination possibly via the PINK1-PARKIN pathway, and ER ubiquitination by mechanisms yet to be identified. p62 activates NRF2 to increase the expression of antioxidant genes, which helps to mitigate APAP-induced reactive oxygen species (ROS) production. YTK-2205 targets the ZZ domain and promotes the recruitment of p62 to the ubiquitinated mitochondria and ER as well as APAP-adducts for selective autophagy to remove APAP-adducts and damaged mitochondria and ER, resulting in decreased hepatocyte necrosis and liver injury. p62 inhibits hepatic macrophage activation by unknown mechanisms, which suppresses hepatic VWF and platelet activation, resulting in deceased liver regeneration in the late recovery phase of AILI. ?: mechanisms not known yet.

In summary, it appears that the role of p62 is very complex, and targeting p62 depends on the different stages of AILI. p62 protects against the early phase of AILI, likely by promoting the removal of APAP-adducts and damaged mitochondria as well as through NRF2 activation. However, p62 impairs the late phase of liver repair/regeneration by increasing hepatic coagulation in AILI (Figure 1). Since most AILI patients have already passed the injury phase, future studies for targeting p62-mediated blood coagulation activation in the late liver regeneration phase would be more clinically relevant.

## 3. Targeting TFEB for AILI

Autophagic degradation relies on the lysosome, which is the terminal component of autophagy, containing more than 50 acid hydrolases. When autophagy is induced, increased lysosomal biogenesis is necessary to meet the need for fusion with autophagosomes and subsequent autophagic degradation. The transcription regulation of lysosome biogenesis genes is mediated by TFEB, which is a vital helix–loop–helix leucine zipper transcription factor belonging to the coordinated lysosomal expression and regulation (CLEAR) gene network [28,66]. Recent studies have shown that TFEB also regulates mitochondrial biogenesis by directly regulating the expression of peroxisome proliferator-activated receptor gamma coactivator-*1* alpha (PGC-1α), a key transcription coactivator for mitochondrial biogenesis [29,30]. TFEB is mainly regulated at its post-translational level via phosphorylation of specific amino acid residues. Several kinases, including the extracellular signal-regulated kinase 2 (ERK2), mTORC1, AKT, GSK3β, and protein kinase Cβ (PKCβ), phosphorylate TFEB at different sites [28,66–68]. TFEB is phosphorylated at Ser142 and Ser211 by mTORC1 and ERK2, which causes it to bind to the cytosolic chaperone 14–3-3 and inactivate in the cytosol [28,67]. Conversely, calcineurin dephosphorylates TFEB at Ser142 and Ser211, promoting its nuclear translocation in response to lysosomal Ca^2+^ release [69].

Our recent findings reveal a new autophagic flux scenario in mouse livers and pancreases after chronic plus binge alcohol treatment [29,70]. This scenario, which we have termed as insufficient autophagy, is characterized by impaired TFEB. Despite an increase in autophagic flux, insufficient autophagy conditions arise due to decreased TFEB-mediated lysosomal biogenesis. The limited number of lysosomes is not sufficient to fuse with all autophagosomes, resulting in a failure to reach full degradation capacity. The pharmacological or genetic activation of TFEB protects against alcohol-induced hepatitis and pancreatitis in mice [29,70]. Since the pharmacological activation of autophagy by mTORC1 inhibitors protects against AILI in the cotreatment and post-treatment of APAP mouse models [20,23] and inhibition of mTORC1 activates TFEB, it is conceivable to speculate that the activation of TFEB should also be beneficial for AILI.

We recently demonstrated that the hepatic levels of TFEB decreased in mouse livers at 6 and 24 h after APAP administration. Liver-specific deletion or overexpression of TFEB exacerbated or protected against AILI, respectively [71]. The activation of hepatic TFEB may protect against AILI via multiple mechanisms. First, the activation of TFEB increases lysosomal biogenesis, resulting in increased clearance of APAP-AD. Second, the overexpression of TFEB increases the hepatic expression of *Sqstm1*, leading to p62-mediated noncanonical NRF2 activation, and accelerates GSH resynthesis after an APAP overdose. Third, the overexpression of TFEB increases PGC1α and mitochondrial transcription factor A (TFAM) as well as the expression of a group of mitochondrial genes, supporting the possible role of increased mitochondrial biogenesis in protecting against AILI [71].

AILI is often associated with hepatic JNK activation. Interestingly, it appears that p62-mediated selective autophagy may not be critical as p62 deficiency does not affect JNK activation [58]. On the other hand, the overexpression of TFEB inhibits APAP-induced JNK activation, suggesting that only the activation of selective autophagy is not required for inhibiting JNK activation [71]. Although there is currently no direct evidence available to explain why the overexpression of TFEB inhibits APAP-induced JNK activation, it is likely that increased mitochondria biogenesis to maintain a healthy pool of hepatic mitochondria may be involved. This is supported by the fact that TFEB also increases the expression of peroxisome proliferator-activated receptor-γ coactivator 1-α (PGC1α), a key transcription coactivator for mitochondria biogenesis.

While inhibiting mTORC1 can increase the activation of TFEB and autophagy, it may also hinder the synthesis of proteins and lipids, which are essential for cell proliferation. Therefore, inhibiting mTORC1 may not be an ideal approach for the late phase of AILI, which requires robust anabolic processes and hepatocyte proliferation. Studies have shown that mice with a genetic deletion of mTORC1 components do not show any protection against AILI due to impaired hepatocyte proliferation and liver regeneration [72]. Thus, it is crucial to search for activators of autophagy and TFEB that are independent of mTORC1 to address this issue. To identify more novel mTOR-independent TFEB agonists, we established a cell-based imaging, high-throughput screening for TFEB agonists using an FDA-approved library. We identified a group of TFEB agonists. Among them, we showed that the cotreatment of APAP with salinomycin, an antibacterial agent, activated TFEB and protected against AILI. Salinomycin treatment also markedly decreased both hepatic and serum APAP-AD and inhibited hepatic JNK activation without affecting mTORC1 activity. However, unpublished data from our lab showed that salinomycin lost its protection if it was given 2 h post-APAP administration, suggesting salinomycin may not be suitable for treating AILI patients. Moreover, as the protective effects of salinomycin against APAP were not validated in TFEB KO mice, it is also possible that other off-target mechanisms may contribute to the protective effects of salinomycin. Also, the mechanisms of how salinomycin activates TFEB remain to be determined in the future.

Narirutin (NR), a key bioactive component from a traditional Chinese medicinal herb, activates hepatic TFEB via enhanced calcineurin activity and protects against AILI in mice [73]. NR also significantly reduces hepatic levels of APAP-AD and oxidative stress but does not affect APAP-induced JNK activation. Importantly, NR exhibits protective effects against AILI even when administered 1 h after APAP injection in mice. The protective effects of NR are lost in liver-specific TFEB KO mice, suggesting that the protection against AILI with NR is mainly mediated by TFEB [73]. Overall, NR shows promise as an mTORC1-independent TFEB activator for treating AILI.

**Figure 2.**
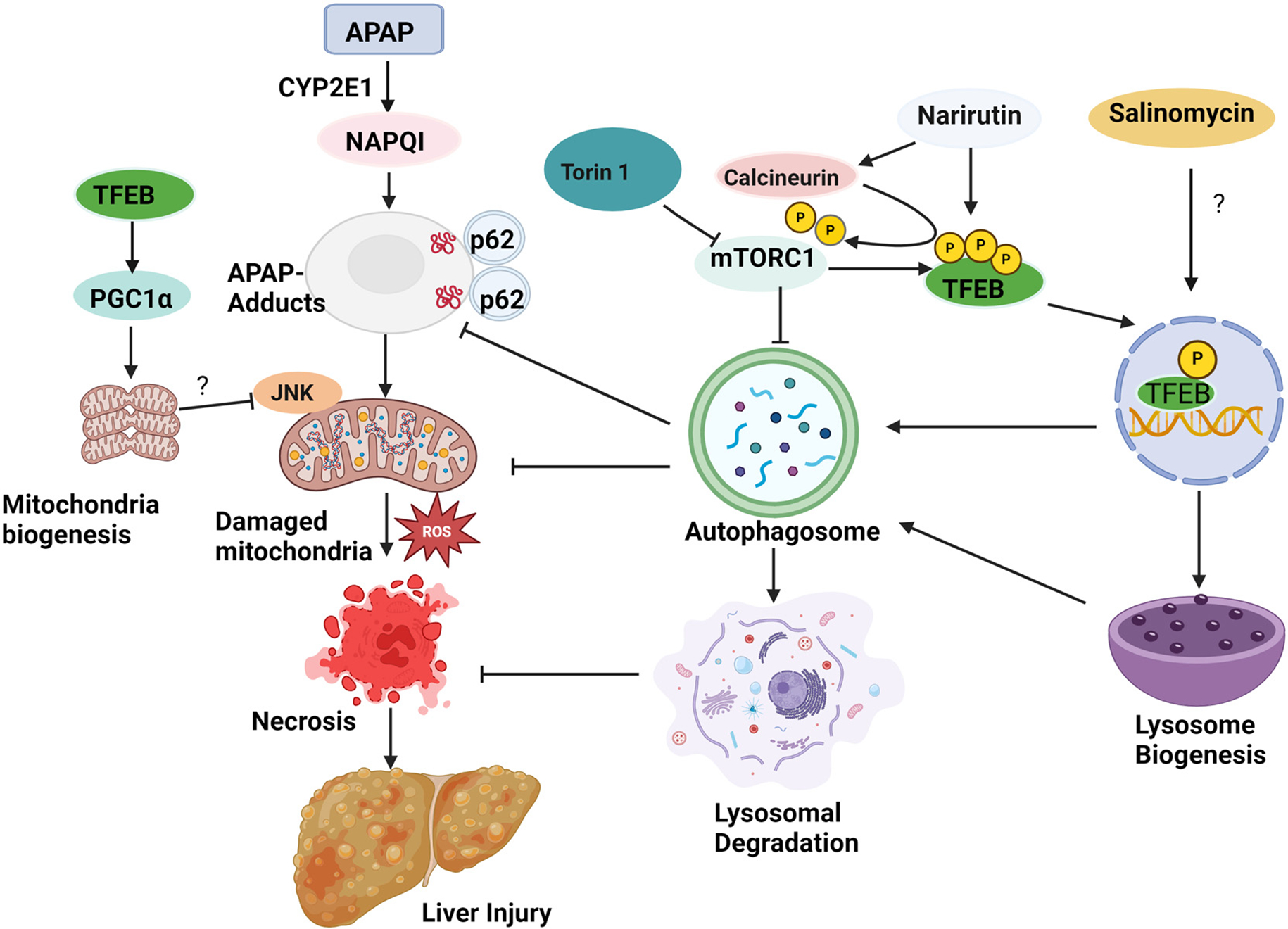
Induction of autophagy protects against acetaminophen-induced liver injury by removing APAP-adducts and damaged mitochondria. ?: mechanisms not known yet.

The inhibition of mTORC1 by a pharmacological inhibitor (Torin 1) induces TFEB activation resulting in increased autophagy and lysosomal biogenesis. Narirutin activates phosphatase calcineurin to increase TFEB dephosphorylation and activation. Salinomycin also activates TFEB by unknown mechanisms. TFEB activation increases lysosomal biogenesis and autophagy which help to remove APAP-adducts and damaged mitochondria, likely via p62, resulting in decreased mitochondrial ROS production and hepatocyte necrosis. The activation of TFEB also leads to increased PGC1α expression, resulting in increased mitochondrial biogenesis that may inhibit JNK activation. Therefore, the pharmacological activation of TFEB may be a promising approach for treating AILI (Figure 2).

## 4. Conclusions and Future Perspectives

In summary, the evidence suggests that autophagy plays a crucial role in removing hepatic APAP-adducts and damaged mitochondria, which are key factors in APAP hepatotoxicity. Therefore, targeting autophagy machinery and lysosomal degradation shows promise for treating AILI. Emerging evidence supports that targeting p62-mediated selective autophagy and TFEB-mediated lysosomal biogenesis are potential therapeutic strategies for AILI. While the overexpression of both p62 and TFEB may be oncogenic, this may not be a concern for the acute liver failure of AILI patients. Further studies should be conducted to identify peptides that enhance p62 LIR-mediated selective autophagy for APAP-adducts and mitochondria, as well as to explore TFEB agonists independent of mTORC1 using high-throughput assays, either imaging-based or luciferase-based, for the TFEB promoter. In addition to the FDA-approved drug library, other small molecule/chemical libraries, including nature products and new synthetic chemicals, should be performed to identify more agonists for autophagy; p62 and TFEB can be tested in experimental AILI models. Moreover, more efforts should be put towards the late phase of liver repair/regeneration rather than the early injury phase when testing these p62 and TFEB agonists, which is more clinically relevant.

## Data Availability

No new data were created or analyzed in this study. Data sharing is not applicable to this article.
